# Trends in inequality in maternal and child health and health care in Uganda: Analysis of the Uganda demographic and health surveys

**DOI:** 10.1186/s12913-022-08630-x

**Published:** 2022-10-20

**Authors:** Alex Ayebazibwe Kakama, Robert Basaza

**Affiliations:** 1 Bishop Stuart University, Mbarara, P.o Box 09, Uganda; 2 Gudie Incubation Centre, Kira Municipality, Kira, Uganda; 3 Gudie Leisure Farm, Masters of Public Health Leadership Program, Uganda Christian University, Mukono, Uganda

**Keywords:** Health inequalities, Health disparities, Maternal and child health

## Abstract

**Background:**

Uganda has made great strides in improving maternal and child health. However, little is known about how this improvement has been distributed across different socioeconomic categories, and how the health inequalities have changed over time. This study analyses data from Demographic and Health Surveys (DHS) conducted in 2006, 2011, and 2016 in Uganda, to assess trends in inequality for a variety of mother and child health and health care indicators.

**Methods:**

The indicators studied are acknowledged as critical for monitoring and evaluating maternal and child health status. These include infant and child mortality, underweight status, stunting, and prevalence of diarrhea. Antenatal care, skilled birth attendance, delivery in health facilities, contraception prevalence, full immunization coverage, and medical treatment for child diarrhea and Acute Respiratory tract infections (ARI) are all health care indicators. Two metrics of inequity were used: the quintile ratio, which evaluates discrepancies between the wealthiest and poorest quintiles, and the concentration index, which utilizes data from all five quintiles.

**Results:**

The study found extraordinary, universal improvement in population averages in most of the indices, ranging from the poorest to the wealthiest groups, between rural and urban areas. However, significant socioeconomic and rural-urban disparities persist. Under-five mortality, malnutrition in children (Stunting and Underweight), the prevalence of anaemia, mothers with low Body Mass Index (BMI), and the prevalence of ARI were found to have worsening inequities. Healthcare utilization measures such as skilled birth attendants, facility delivery, contraceptive prevalence rate, child immunization, and Insecticide Treated Mosquito Net (ITN) usage were found to be significantly lowering disparity levels towards a perfect equity stance. Three healthcare utilization indicators, namely medical treatment for diarrhea, medical treatment for ARI, and medical treatment for fever, demonstrated a perfect equitable situation.

**Conclusion:**

Increased use of health services among the poor and rural populations leads to improved health status and, as a result, the elimination of disparities between the poor and the wealthy, rural and urban people.

**Recommendation:**

Intervention initiatives should prioritize the impoverished and rural communities while also considering the wealthier and urban groups.

## Introduction

Equity is one of the basic principles of Primary Health Care, and it is reflected in most countries’ health policy [1]. Despite governments’ commitment to promoting pro-poor health policies and interventions, levels of disparity in health status and utilization of basic health care interventions remain high throughout Sub-Saharan Africa [2]. Evidence suggests that in Sub-Saharan African countries, healthcare consumption is determined not by need, but by wealth, geographical location, education, and individuals’ ability to pay [3]. Access to healthcare tends to follow the ‘inverse care law,‘ which supposes that the wealthy, who have a lower need for healthcare, have better access to quality care, while the poor and marginalized, who have a greater need, have limited access to quality care [4]. In South Africa, the impoverished have worse health than the wealthy [5]. Similarly, in Uganda, the poor are sicker and have less access to publicly funded health services than their wealthier counterparts [6]; and household welfare status has been highlighted as a critical predictor of health inequalities [7].

Inequalities, disparities, and inequities are commonly used interchangeably [8, 9], although all refer to discrepancies in the utilization or access to services by various groups. Health disparities are differences in the use or access to healthcare services that are unnecessary, preventable, unfair, and unjust [10]. Equity in healthcare refers to ensuring that all individuals have access to a minimum standard of treatment based on need rather than any other factor, such as financial position, geographical location, or capacity to pay [10].

The aim of this study was to analyze equity trends in Uganda for several indices of maternal and child health and healthcare, utilizing data from the Uganda Demographic and Health Surveys of 2006, 2011, and 2016. Specifically, the study assessed the differences in absolute percentage changes in population averages between the wealthiest and poorest, rural and urban populations; calculated the quintile ratios and concentration indices to determine the magnitude and trend of inequalities and identified variables that displayed improving or worsening trends of inequality.

Monitoring the levels and trends in health inequalities and health service utilization is critical for intervention programs that allocate finite public resources to people who are disadvantaged and have greater needs. The findings of this study may be used by other researchers in the design of future investigations. The findings can help policymakers and technocrats establish relevant health policies and initiatives that promote health equity. Ugandan communities might gain because subsequent policies and actions shall become more responsive to local needs.

## Statement of the problem

In the previous few decades, Uganda has made significant progress in improving maternal and child health [11]. However, little is known about how this development has been distributed among different socioeconomic groups, and how health inequalities have changed over time. In Uganda, research on health disparities is scarce. The majority of studies on health inequalities have concentrated on a single health indicator, and none have examined trends in inequality. For instance, Robertson et al. [12] investigated urban-rural discrepancies in Chronic Obstructive Pulmonary Disease (COPD) management and access in Uganda. Also, Ssewanyana & Kasirye [7] used data from Uganda demographic and health surveys to study the factors of child nutrition status in Uganda. Using data from three successive Demographic and Health Surveys (DHS) from 2006, 2011, and 2016, this study assesses the degree and trends in disparities for a variety of indicators of maternal and child health and health care for varied socioeconomic categories, rural and urban populations.

## Methodology

### Study settings

Uganda is a landlocked country in East Africa with a total land area of 241,559 square kilometers and a population of 44.3 million people [13]. Uganda is classified as a low-income country by the World Bank, with a low human development index (HDI of 0.544), ranking 159th out of 189 countries [13]. Up to 21.4% of the population lives in poverty, with less than US$ 1 per person per day [14]. Overall health expenditure per capita is $ 43, and total health expenditure accounts for 6.5% of GDP [15]. By the year 2019, infant mortality and under-five mortality were rated at 33 and 46 per 1000 live births respectively; and maternal mortality ratio was at 375 deaths per 100,000 live births [16].

### Study variables

The dependent variable in this study was Inequality levels; measured through quintile ratios and concentration indices. *Quintile ratio* provides information on the disparities between the wealthy and the poor [17]. It allows for comparisons of health status or health-care utilization between the richest and poorest quintiles. The *Concentration Index* on the other hand, is a relative measure of inequality that reveals how concentrated a health indicator is among the disadvantaged or advantaged [17]. It uses data from all five wealth quintiles to provide a complete picture by quantifying the degree of inequalities in the population.

Explanatory variables were grouped into two categories: Maternal and child health variables, such as infant mortality rate, under-five mortality rate, and child underweight, Child stunting, prevalence of anemia, prevalence of fever, prevalence of Acute Respiratory tract Infections (ARIs), prevalence of diarrhea; in children under five years of age, and Mother’s low Body Mass Index (BMI). The other category includes variables of healthcare service use, such as antenatal care, skilled birth attendance, delivery in health facilities, full immunization coverage, medical treatment for child diarrhea, contraceptive prevalence rate, deliveries in government-owned health facilities, medical treatment for ARIs, medical treatment for fever in children under the age of five, and use of ITN.

### Sources of data and sampling procedures

This study makes use of data from three Uganda Demographic and Health Surveys (UDHS), which were conducted in 2006, 2011, and 2016 by a state specialized unit, Uganda Bureau of Statistics (UBOS). The UDHS uses a stratified two-stage cluster sampling procedure. In the first stage, clusters are selected from sampling frames using the most recent census. Households are selected from each cluster at the second stage. The UDHS captures information in such areas as: women’s and children’s demographic and socioeconomic characteristics, household characteristics, maternal and child health status parameters, and maternal and child healthcare service parameters using questionnaires. It also involves conducting height and weight measurements of children and women, testing for anemia, malaria and Vitamin A deficiency [11, 18, 19].

Inclusion criteria: All women age 15–49 and who were either permanent residents of the selected households or visitors who stayed in the household the night before the survey were eligible to be interviewed. In one-third of the sampled households, all men age 15–54, including both usual residents and visitors who stayed in the household the night before the interview, were eligible for individual interviews. In the subsample of households selected for the male survey, anaemia testing was performed among eligible women age 15–49 and men age 15–54 who consented to being tested and among children age 6–59 months whose parents or guardians consented. In the same subsample, blood samples were collected from children age 6–59 months whose parents or guardians consented to malaria testing with rapid diagnostic test (RDT) kits. Height and weight measurements were recorded for children age 0–59 months, women age 15–49, and men age 15–54. In 2006, there were 8,531 women and 2,503 men interviewed; in 2011, there were 8,674 women and 2,295 men interviewed; and in 2016, there were 18,506 women and 5,336 males interviewed. Up to 10,173 children under five years (2,687 children in 2006, 2,350 children in 2011 and 5,136 children in 2016) participated in the nutrition assessment exercise [11, 18, 19]. The surveys excluded households in institutional living arrangements such as army barracks, hospitals, police camps, and boarding schools [11, 18, 19].

### Theoretical framework

This study adopted a structural theory to understanding health inequalities [20]. The structural theory suggests that differences in social groups’ socioeconomic circumstances, such as income, wealth, power, environment, and access, explain differences in health outcomes [21]. This argument is supported by evidence that health inequalities have decreased when structural inequalities have decreased [22] and that community health has improved when more resources have been provided [23], and, most convincingly, that the people with the most resources in any society are always the healthiest, regardless of their behaviors [24]. Even when a health issue is obviously linked to a genetic mutation, mortality disparities by socioeconomic class are large [25].

### Measurement of variables

Indicators analyzed are in two categories: Maternal and child health outcome indicators, such as infant mortality rate, under-five mortality rate, and child underweight, Child stunting, prevalence of anemia, prevalence of fever, prevalence of Acute Respiratory Tract Infections (ARIs), prevalence of diarrhea; in children under five years of age, and Mother’s low BMI.

#### Height and weight measurement

This study considered children with a Z-score less than minus two standard deviations (SD) from the median of the WHO reference population for height-for-age (Stunting) and weight-for-age (Underweight) [26; 27]. The UDHS collected data on children’s nutritional status by measuring the height and weight of all children under the age of five in a subsample of one in every three families chosen for the survey. Weighing was done with a lightweight electronic SECA scale designed and built under the supervision of UNICEF. Shorr Productions designed a measuring board that was used to take height measurements. Children under the age of 24 months were measured laying down (recumbent length) on the board, while older children were measured standing tall [19]. The nutritional status of children was determined using WHO’s new growth guidelines published in 2006 [27].

#### Mother’s BMI levels

A BMI of 18.5 was utilized in this study to identify thinness or acute malnutrition in women aged 15 to 49 [28]. BMI is calculated by dividing one’s weight in kilograms by one’s height in meters squared (kg/m^2^). The body mass index (BMI) is used to determine whether a person is lean or obese. The height and weight of women aged 15 to 49 were measured in one out of every three UDHS homes [18].

#### Anaemia screening

In this study, anemia was defined as a haemoglobin level in children less than 11 g/dl [29]. Blood samples were collected for anaemia testing from eligible women and men who consented to be examined, as well as from all children aged 6–59 months who had permission from their parents or guardians. A drop of blood was taken from the prick site (a finger prick or a heel prick in the case of children age 6–11 months) into a microcuvette, and haemoglobin analysis was performed on-site using a battery-powered portable HemoCue analyzer [19].

#### Malaria screening

Malaria testing was only done on children aged 6 to 59 months; no adults were screened. A drop of blood was tested immediately using the SD Bioline Pf/Pv RDT, which is a qualitative test for the detection of histidine-rich protein II (HRP-II) antigen of Plasmodium falciparum (Pf) and/or Plasmodium vivax (Pv) in human whole blood, using the same finger (or heel) prick used for anaemia testing [19]. Plasmodium falciparum is the most common Plasmodium species in Uganda.

### Data analysis

Inequality was measured using two different methods: First, we considered quintile ratios. The ratio indicator compares health status or health-care utilization between the richest and poorest quintiles. To some extent, this indicator provides information on the disparities between the wealthy and the poor. However, it is based solely on data from the two wealthiest quintiles and ignores the remaining three quintiles between the top and bottom, and hence cannot provide a comprehensive picture of inequality over the entire population [30].

The second indicator is the Concentration Index; which is a relative measure of inequality that reveals how concentrated a health indicator is among the disadvantaged or advantaged [17; 30]. Its size represents the degree of inequality. The concentration index calculates the degree of economic inequality by utilizing data from all five quintiles. As a result, it is a synthesis of inequality throughout the entire population [17]. The concentration index has a range of -1 to + 1. Traditionally, if the health status measure is a “bad” in the sense that it depicts poor health, the index takes a negative value, suggesting that the poorest segments of the population bear the largest burden of poor health. If the health status measure is a “good,“ in the sense that it indicates a positive feature of health, the index takes a positive value, suggesting that the poor are significantly less healthy. In the absence of inequities, the concentration index has a value of zero. The concentration index (C) is calculated in a spreadsheet program from grouped data using the following formula [17].

### C= (P1L2-P2L1) + (P2L3-P3L2) +… + (PT-1 LT-PTLT-1)

Where P*t* is the cumulative percent of the sample ranked by wealth status in group *t*, L*t* is the corresponding Lorenz curve ordinate, and T is the total number of wealth groups, which is five in this analysis [17].

## Results

This section begins with demographic characteristics of participants, followed by findings on maternal and child health outcomes and ends with results on health-care utilization indicators.

### Demographic characteristics of participants

A total of 5928 children between the ages of 12 and 23 months, 29,691 children under the age of five, 9642 women, and 9229 men between the ages of 15 and 49 were interviewed. A total of 31,769 live births documented in the five years preceding the surveys were considered. Table 1 holds details on the survey participants.

**Table 1:** Demographic characteristics of participants in the surveys

	Gender		Residence		Wealth quintile				
	**Male**	**Female**	**Urban**	**Rural**	**Lowest**	**Second**	**Middle**	**Fourth**	**Highest**
Children 12–23 months	2969	2959	1030	4898	1269	1305	1150	1077	1127
Children under five years.	14,774	14,917	5055	24,636	6626	6354	5844	5366	5501
Women aged 15–49 years of age		9642	2150	7492	1614	1709	1827	2051	2441
Men aged 15–49 years	9229		1969	7260	1535	1767	1749	2020	2158
Live births in the five years preceding the survey			5333	26,436	7151	6830	6242	5764	5782

### Inequalities in maternal and Child Health status

This sub-section presents population averages, quintile ratios and concentration indices for health outcome indicators for all wealth quintiles, rural and urban populations. Table 2, summaries the data.

All indicators showed universal improvement across quintiles, and between rural and urban populations. The most success was made in lowering infant and under-five mortality. Between 2006 and 2016, the infant mortality rate fell from 76 to 43 deaths per 1,000 live births, and the under-five mortality rate fell from 137 to 64 deaths per 1,000 live births [11, 18, 19]. All other health status markers showed a similar pattern, as displayed in Table 2.

**Table 2:** Health status metrics and associated quintile ratios, and concentration indices

	Geographical location				Wealth Quintiles							
**Health indicators**		**Rural**	**Urban**	**Low/High Ratio**	**Lowest**	**Second**	**Middle**	**Fourth**	**Highest**	**Total**	**Low/High Ratio**	**Concentration Index (CI)**
Infant Mortality rate (per 1000 live births)	2006	88	68	**1.3**	102	92	87	80	63	**76**	**1.6**	-0.09
	2011	66	54	**1.2**	76	69	64	63	48	**54**	**1.6**	-0.08
	2016	57	36	**1.6**	56	50	44	48	39	**43**	**1.4**	-0.05
Under-five mortality rate (per 1000 live births)	2006	153	114	**1.3**	172	157	155	140	108	**137**	**1.6**	-0.08
	2011	111	77	**1.4**	123	125	100	104	72	**90**	**1.7**	-0.10
	2016	68	52	**1.3**	88	79	73	69	53	**64**	**1.7**	-0.09
Stunting among children under age 5 (%)	2006	39.5	25.5	**1.5**	43.4	38.0	44.4	37.6	24.3	**38**	**1.8**	-0.07
	2011	35.6	18.6	**1.9**	37.3	30.9	45.0	30.5	20.8	**33.4**	**1.8**	-0.07
	2016	30.2	23.5	**1.3**	32.3	33.2	33.0	27.2	16.7	**28.9**	**1.9**	-0.09
Underweight among children under age five (%)	2006	16.5	10.6	**1.6**	20.6	15.6	17.0	16.5	8.4	**15.9**	**2.5**	-0.07
	2011	14.9	6.6	**2.3**	18.1	14.3	17.3	9.5	8.4	**13.8**	**2.2**	-0.14
	2016	11.2	7.5	**1.5**	15.0	11.5	11.6	8.6	4.4	**10.5**	**3.4**	-0.19
Anemia among children under age five (%)	2006	74.3	56.6	**1.3**	79.7	74.8	73.3	72.3	60.5	**72.6**	**1.3**	-0.04
	2011	50.9	38	**1.3**	59	51.7	51	42.8	38.2	**49.3**	**1.5**	-0.08
	2016	54	47.7	**1.1**	65.6	54.4	48.7	48.5	44.8	**52.8**	**1.5**	-0.08
Diarrhea among children under age five (%)	2006	26.5	19.7	**1.3**	33.7	27.2	23.6	23.6	18.1	**25.8**	**1.9**	-0.11
	2011	23.7	21.8	**1.1**	28.8	25.2	21.8	20.6	19.5	**23.4**	**1.5**	-0.08
	2016	20.2	17	**1.2**	22.2	21	19.2	18.1	16.5	**19.5**	**1.3**	-0.05
ARI among children under age five (%)	2006	15	10.7	**1.4**	18.7	16.8	13.8	12.2	8.9	**14.5**	**2.1**	-0.13
	2011	15.2	13	**1.2**	20.1	16.5	12.6	12.1	11.9	**14.8**	**1.7**	-0.11
	2016	10.1	7.1	**1.4**	12.7	10.5	9	8.3	5.5	**9.3**	**2.3**	-0.14
Fever among children under age five (%)	2006	43	25	**1.7**	48.3	44.7	37.1	39.2	32.5	**40.9**	**1.5**	-0.06
	2011	42.1	30.3	**1.4**	49.8	42.6	36.8	40.7	30.3	**40.4**	**1.6**	-0.09
	2016	36.4	22	**1.7**	43.9	37	32.6	31.2	19.6	**33.3**	**2.2**	-0.13
Mothers’ low BMI	2006	13.5	5.9	**2.3**	23.2	15.1	12.3	8.6	5.9	**12.1**	**3.9**	-0.26
	2011	12.9	7.6	**1.7**	22.8	18.3	9	7.6	5.9	**11.7**	**3.9**	-0.29
	2016	9.3	6.9	**1.3**	16.9	10.9	8.9	5.4	4.3	**8.7**	**3.9**	-0.28

#### Changes in absolute terms

**Fig. 1:**
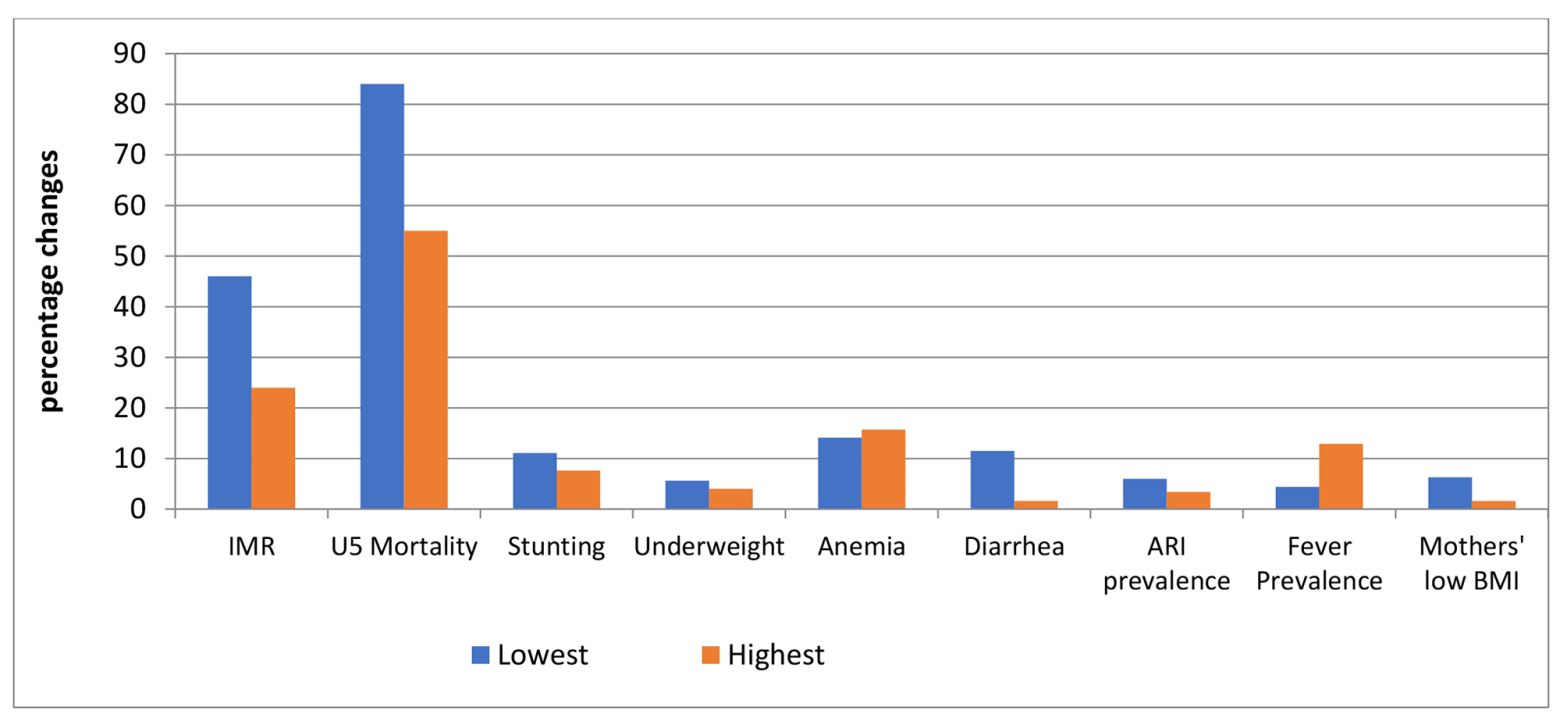
Absolute percentage changes comparing different socioeconomic status (lowest and highest quintiles)

The poorest group improved more than the richest. For example, the lowest quintile’s newborn mortality rate decreased by 45%, while the richest quintile’s rate decreased by 38%. Similarly, stunting decreased by 11% points in the lowest quintile compared to 8% points in the richest quintile. However, the lowest quintile group improved less than the richest group in terms of anaemia and fever prevalence. Changes in other health status metrics are as shown in Fig. 1.

Similarly, the rural population improved at a faster rate than the urban population. Under-five mortality, for example, fell by 85 deaths per 1000 live births in rural areas compared to 62 deaths per 1000 live births in urban areas. Stunting prevalence decreased by 9% points in rural areas but only by 2% points in urban areas.

#### Quintile ratios

Data analysis utilizing quintile ratios reveals that there is inequality between the poorest and richest quintiles. All of the variables have quintile ratios greater than one, showing the presence of inequalities that favor the wealthy over the poor. Furthermore, the quintile ratios rose with time in most indices, showing a growing inequality gap between the rich and the poor.

The ratios of the lowest (poorest) to highest (wealthiest) quintiles, as well as rural to urban ratios, are shown in Table 2.

The greatest ratios, and therefore bigger disparities, were reported in the under-five mortality rate, child stunting, underweight in children, prevalence of anaemia in children, prevalence of ARI, prevalence of fever, and mothers with low BMI. For example, the frequency of underweight in children among the poorest was 2.5 times greater in 2006 than among the wealthiest, and 3.4 times higher in 2016. A similar pattern may be seen for the prevalence of fever in children, the prevalence of ARI, the prevalence of anaemia in children, and the prevalence of child stunting. The prevalence of low BMI among mothers remained extraordinarily high, at 3.9 across the board.

Except for infant mortality rate, all indices show dropping quintile ratios for rural vs. urban populations from 2006 to 2016. This finding depicts shrinking disparities between rural and urban populations.

#### Concentration indices

First, all of the concentration indices for health outcome indicators were negative, indicating that the poor are more afflicted by illness. Second, with the exception of the infant mortality rate and the incidence of diarrhea, all indicators showed increasing concentration indices.

For example, the concentration index for childhood underweight grew from − 0.07 to 2006 to -0.19 in 2016. Rising concentration indices suggest that inequality gaps are widening, against persons with low socioeconomic status.

### Inequalities in the utilization of maternal and Child Health Services

At the population level, there has been a consistent and significant increase in all measures of service consumption, for all quintile groups, rural and urban populations. For example, the proportion of births attended by a skilled birth attendant increased from 42% to 2006 to 74% in 2016. Contraceptive prevalence increased from 18% to 2006 to 35% in 2016. Table 2 and.

**Fig. 2:**
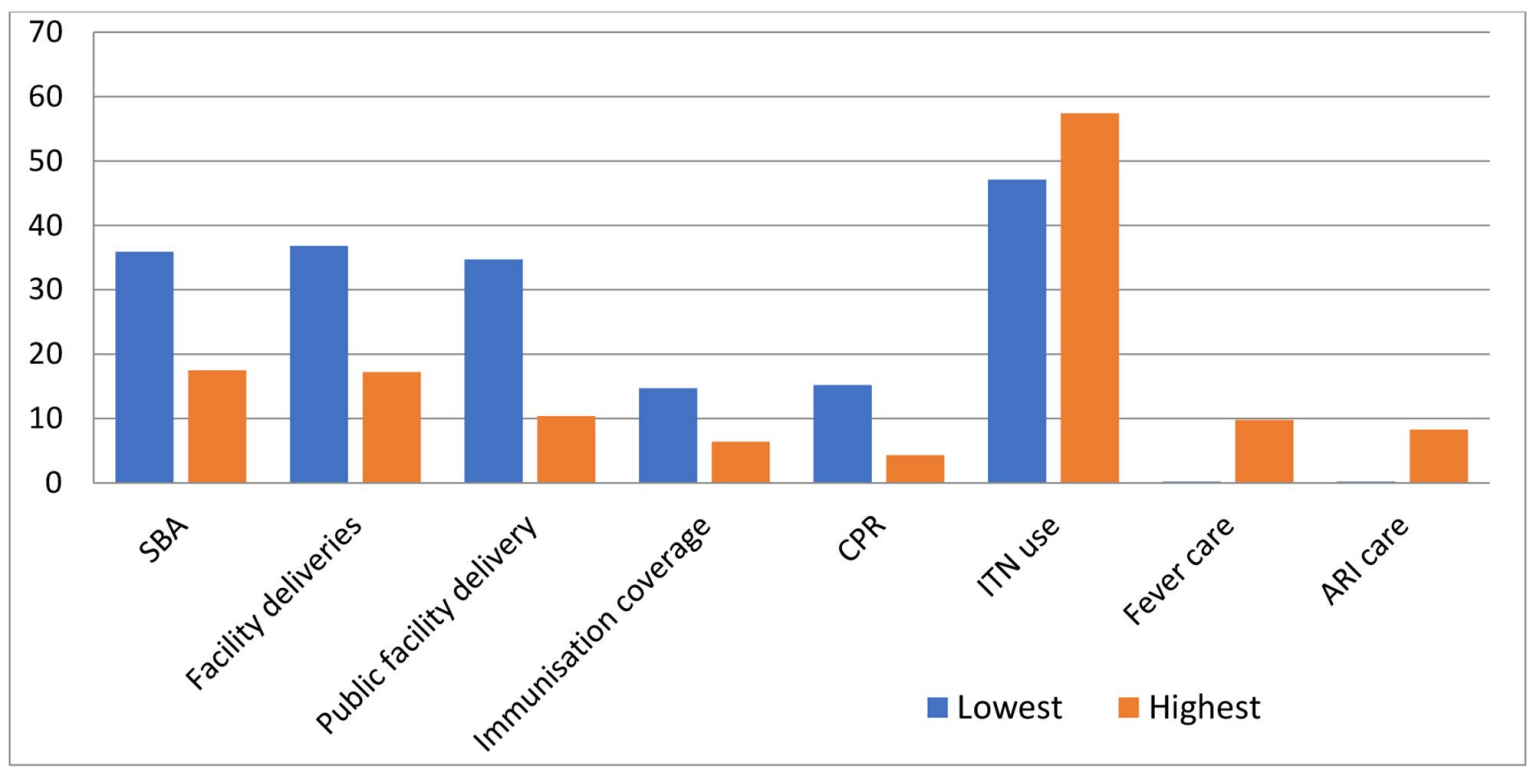
Absolute percentage point increases in health-care utilization between richest and poorest quintiles

Figure 2 depict graphical representations of absolute changes for socioeconomic quintiles and rural vs. urban areas, respectively.

All indicators of Maternal and Child Health Services utilisation showed universal improvement across quintiles, and between rural and urban populations. The most success was made in increasing the proportion of births attended by a skilled birth attendant, ITN usage, immunization coverage, and contraceptive use. For example, the proportion of births attended by a skilled birth attendant increased from 42% to 2006 to 74% in 2016. Contraceptive prevalence increased from 18% to 2006 to 35% in 2016. All other indicators showed a similar pattern, except proportion of Children under five years with fever that received medical treatment which reduced from 74.7 to 48.4% as displayed in Table 3.

**Table 3:** Health service utilization metrics and associated quintile ratios, and concentration indices

					Wealth Quintile							
**Health indicators**		**Rural**	**Urban**	**Urban/Rural Ratio**	**Lowest**	**Second**	**Middle**	**Fourth**	**Highest**	**Total**	**High/Low Ratio**	**Concentration Index (CI)**
Births assisted by skilled birth attendant (%)	2006	37.3	80	**2.1**	28.4	31.7	35	49.1	76.6	**42.1**	**2.7**	0.19
	2011	52.8	89.1	**1.7**	43.5	48.9	54.4	59.6	88.4	**58**	**2.0**	0.12
	2016	70	89.9	**1.3**	64.3	64.3	71.7	79.3	94.1	**74.2**	**1.5**	-0.72
Births delivered in health facility (%)	2006	36.3	78.7	**2.2**	27.4	30.9	33.9	47.9	75.5	**41.1**	**2.8**	0.20
	2011	52	89.5	**1.7**	42.2	48.9	54.4	58.4	87.7	**57.4**	**2.1**	0.12
	2016	69.5	87.8	**1.3**	64.2	63.1	70.7	79	92.7	**73.4**	**1.4**	-0.72
Births delivered in government health facility (%)	2006	25.6	57	**2.2**	21.3	23.1	25.1	32.2	50	**29.1**	**2.3**	0.17
	2011	40.8	63.5	**1.6**	37.2	39.1	43.6	42.7	59.7	**44**	**1.6**	0.08
	2016	55.7	63.2	**1.1**	56	53.1	58	59.7	60.4	**57.3**	**1.1**	-0.78
Children 12–23 months fully immunized (%)	2006	45.7	51.1	**1.1**	41.4	45.0	48.2	49.3	47.9	**46.2**	**1.2**	0.03
	2011	50.2	60.8	**1.2**	50.6	51.4	48.7	52.6	54.9	**51.6**	**1.1**	0.01
	2016	54.5	55.5	**1.0**	56.1	54.7	55.9	55.2	54.3	**55.2**	**1.0**	-0.77
Children under five years with diarrhea that received medical treatment (%)	2006	70.3	68.9	**1.0**	77.6	69.6	61.3	67.8	70.5	**70.2**	**0.9**	-0.03
	2011	72.7	70.2	**1.0**	73.7	72.5	74.4	72.7	67.1	**72.4**	**0.9**	-0.02
	2016	70.6	70.2	**1.0**	73.5	69.6	69.3	68.2	70.8	**70.5**	**1.0**	-0.85
Contraceptive prevalence rate (%)	2006	15.1	36.5	**2.4**	7.2	12.1	13.1	20.3	37.9	**17.9**	**5.3**	0.09
	2011	23.4	39.2	**1.7**	12.7	21.2	24.7	31.0	39.1	**26.0**	**3.1**	0.20
	2016	33.0	40.7	**1.2**	22.4	32.2	35.9	40.2	42.2	**34.8**	**1.9**	-0.67
Children under five years with ARI, that received medical treatment (%)	2006	73.7	68.3	**0.9**	79.5	67.7	69.8	72.8	77.5	**73.3**	**1.0**	-0.01
	2011	78.4	80.8	**1.0**	77.8	78.9	78.1	77.2	82.3	**78.7**	**1.1**	0.00
	2016	80.0	82.6	**1.0**	79.7	77.6	78.2	84.5	85.8	**80.4**	**1.1**	-0.86
Antenatal visits to a trained personnel (%)	2006	93	97.2	**1.0**	93.2	92.3	92.9	93.2	96.4	**93.5**	**1.0**	0.00
	2011	94.4	97.4	**1.0**	93.9	94.5	94.3	94.5	97.1	**94.9**	**1.0**	-0.08
	2016	97.1	98.1	**1.0**	96.0	96.9	98.3	97.3	98.1	**97.3**	**1.0**	-0.79
Insecticide-treated net (ITN) use in Children under 5 years (%)	2006	8.3	21.3	**2.6**	10.7	9.7	4.9	8.8	15.4	**9.7**	**1.4**	0.04
	2011	41.9	48.9	**1.2**	44.8	40.7	39	41.3	48.6	**42.2**	**1.1**	0.01
	2016	67.0	60.8	**0.9**	57.8	58.2	59.1	64.3	72.8	**62**	**1.3**	-0.77
Children under five years with fever that received medical treatment (%)	2006	74.5	76.8	**1.0**	78.1	72.0	72.0	72.3	80.0	**74.7**	**1.0**	0.00
	2011	79.2	87.2	**1.1**	78.8	79.1	82.3	77.6	84.5	**80.1**	**1.1**	0.01
	2016	55.0	47.3	**0.9**	46.6	45.3	45.3	49.4	62.8	**48.4**	**1.3**	-0.84

#### Changes in absolute terms

For all variables, absolute gains are bigger among the lowest quintile (poorest) than among the wealthiest, with the exception of ITN use among children under five years and fever and ARI treatment for children under five years, as displayed in Fig. 2. The increases in the poorest group for skilled birth attendants at delivery (SBA) and facility deliveries are twice as large as the increases in the wealthiest group.

On a similar note, the rural population experienced greater absolute increases than the urban population in all variables except for diarrhea and ARI treatment. The improvements in rural populations’ usage rates for skilled birth attendants at delivery (SBA), facility deliveries, ITN use, and contraceptive use were at least twice as large as the changes in urban populations’ usage rates.

#### Quintile ratios

Despite significant improvements in health-care utilization among the poorest households, socioeconomic inequities were discovered. This is demonstrated by quintile ratios greater than one, in Table 2. However, the ratios for all variables fell with time, indicating shrinking differences between the richest and poorest populations. The quintile ratio for facility deliveries, for example, declined from 2.8 to 2006 to 1.4 in 2016. A similar pattern can be seen for the contraceptive prevalence rate as well as all other measures.

Despite significant improvements in rural health-care utilization, rural-urban disparities were discovered. This finding was supported by quintile ratios greater than one in the majority of the services, in Table 3. The high quintile ratios suggest that the urban population uses more services than the rural population. However, quintile ratios decreased with time, indicating that inequality was shrinking. The quintile ratio of health facility deliveries, for example, fell from 2.2 in 2006 to 1.3 in 2016.

#### Concentration indices

Except for diarrhoea treatment, all indicators exhibited positive concentration indices in 2006 and 2011, before changing to negative levels in 2016 as displayed in Table 3. This demonstrates a shift from a pro-rich inequality to a more equitable one (pro-poor). For example, facility birth indices fell from 0.20 in 2006 to 0.12 in 2011 to -0.72 in 2016.

## Discussion

### Variables showing widening trends of inequality

The following indicators demonstrated worsening trends in socioeconomic and rural-urban disparities: Rate of under-five mortality (per 1000 live births); Malnutrition in children under the age of five; Anaemia in children under the age of five; Acute Respiratory Tract Infection (ARI) in children under the age of five; Fever prevalence in children under the age of five; and Mothers with a low Body Mass Index (BMI). These trends were evidenced by rising quintile ratios and concentration indices. Previous studies have linked Child mortality to economic inequality rather than epidemiological causes, with the disadvantaged poorer households registering higher mortality rates [31; 32]. Similarly, Wagstaff and Watanabe [33] identified that malnutrition inequalities affect the poor; and it diminishes consistently with growing living standards. Furthermore, Alaofè et al. [34] revealed a higher risk of anaemia in children from low socioeconomic backgrounds, Furthermore Sultana et al. [35] discovered that children from the poorest quintile were 2.36 times more likely to suffer from ARIs. In a similar vein, Filmer, [36] discovered that fever occurrences are frequently lower at the very top of the wealth distribution. Other studies established that maternal under-nutrition was highly related to socioeconomic characteristics; and concentrated among the rural population [37–39].

According to structural theory, the worsening trends in mother and child health can only be reversed if the factors of income disparity are addressed effectively. Previous research indicates that income has a positive impact on child health. For example, Lawson and Appleton [40], identified that doubling household income would dramatically reduce malnutrition and morbidity in pre-school children by 20%. It was also discovered in Vietnam that fluctuations in stunting rates are partly explained by levels of income disparity, implying that income inequality is a primary driver of health inequities [41]. In addition to income, education programs have been proposed as an approach for improving maternal and child health outcomes [42]. Ssewanyana and Kasirye [7] suggest that raising primary school completion rates for mothers reduces Uganda’s infant mortality rate; nonetheless, there is a need to enhance female education beyond the primary level if Uganda is to see meaningful changes in child health status.

### Variables showing narrowing trends of Inequality

The following metrics showed improving trends in socioeconomic and rural-urban differences, indicating a move closer to full parity: Infant Mortality rate (per 1000 live births); Diarrhea prevalence in children under the age of five; The proportion of births attended by skilled birth attendants; The proportion of births that take place in a health facility; Percentage of children aged 12 to 23 months who are fully vaccinated; Diarrhea treatment for children under the age of five; Contraceptive prevalence rate (%); Medical treatment for ARI symptoms in children under the age of five; ITN usage among children under five years of age; and Fever treatment in children under the age of five. The downward trend of inequality was demonstrated by falling quintile ratios and concentration index.

Consistent with this study, Hosseinpoor et al. [43] identified a diminishing socioeconomic disparity trend in India’s infant mortality rate, while Kumar and Singh [44] identified that rural areas had much greater infant mortality rates than metropolitan locations. In addition, Kengia [47] and Asamoah [48] concur that differences in skilled birth attendant utilization between socioeconomic classes and rural-urban groups had greatly narrowed. Barata et al. [50] identified that underprivileged children had higher vaccine coverage than those from the highest socioeconomic strata. Asamoah et al. [52] identified that there is growing equality in the use of modern contraceptives, and that the rural–urban split in modern contraceptive use has virtually evaporated.

On the contrary, Kumi-Kyereme and Amo-Adjei [45, 46] found that wealthy households were more likely than poorer ones to seek medical treatment for childhood diarrhea; and that rural poor had lower odds of reporting diarrhoea than affluent or urban inhabitants. Secondly, Chigwenah [51], show that higher socioeconomic status households are more likely than lower socioeconomic status families to have their children immunized. Janevic et al. [53] discovered, that women in poor communities were less likely than those in wealthier groups to utilize modern contraception. Dagne et al. [54], found that access to COPD therapy was greater in urban areas than in rural areas, and rural living was strongly related with acute respiratory infection. Hasan et al. [49] discovered that institutional delivery services favored the urban-based and rich women. Furthermore, Hasan [55] found that wealthy households were more likely than poor households to seek medical treatment for their children’s fever; and Kanmiki et al. [56] revealed that the wealthy were more likely than the poor to own and utilize ITNs [56].

Overall, healthcare utilization metrics demonstrated decreasing disparities toward a perfect equality position; and a totally flawless equity situation was also observed in indicators linked to medical treatment for diarrhea, ARI symptoms, and fever in children under the age of five. According to structural theory, the achievements can be attributed to structural adjustments; the health sector reforms conducted by Uganda to make services more accessible, particularly to the poor and those living in rural regions [57]. Specific reforms include: Sector-Wide Approach (SWAP) processes [58]; abolishing user fees in government health units, [59]; improved systems in financing and supply of medicines [60]; Public-private partnership (PPP) [61]; Decentralized service [62]; and Improved resource allocations to primary health care services [63], introduction of Poverty Action Fund to channel resources to high priority budget areas[63].

## Conclusion

In majority of the metrics studied, the results show a significant improvement in population averages. The rises are universal, ranging from the lowest to the wealthiest groups, as well as between rural and urban areas. However, significant socioeconomic and rural-urban disparities persist. Under-five mortality, malnutrition in children (Stunting and Underweight), the prevalence of anaemia in children, mothers’ low BMI, and the prevalence of ARI in children were all found to have worsening trends of inequities.

On the other hand, improving or lowering disparity levels toward a perfect equity stance were mostly identified in healthcare use indicators such as skilled birth attendants, facility delivery, contraceptive prevalence rate, child immunization, diarrhea treatment, and ITN use. Three healthcare use measures, namely medical treatment for diarrhea, medical treatment for ARI symptoms, and medical treatment for fever in children under the age of five, demonstrated a perfectly flawless equity situation.

## Data Availability

The datasets analyzed during this study are available in the Uganda Bureau of Statistics (UBOS) repository, available at: https://www.ubos.org/?pagename=explore-publications&p_id=25.

## Abbreviations

ARI: Acute Respiratory tract Infections
SBA: Skilled Birth Attendants
BMI: Body Mass Index
UBOS: Uganda Bureau of Statistics
CDC: Center for Disease Control
UNDP: United Nations Development Program
COPD: Chronic Obstructive Pulmonary Disease
USD: United States Dollars
DHS: Demographic and health Survey
WHO: World Health Organisation
GDP: Gross Domestic Product
HDI: Human Development Index
ITN: Insecticide Treated mosquito Net
